# Supplementary material for: Resolving the topology of encircling multiple exceptional points
Source: Nat Commun. 2024 Feb 14;15:1369. doi: 10.1038/s41467-024-45530-6 (PMC10867139; doi:10.1038/s41467-024-45530-6)
Supplement: Supplementary file 1 — Supplementary Information [file 41467_2024_45530_MOESM1_ESM.pdf]

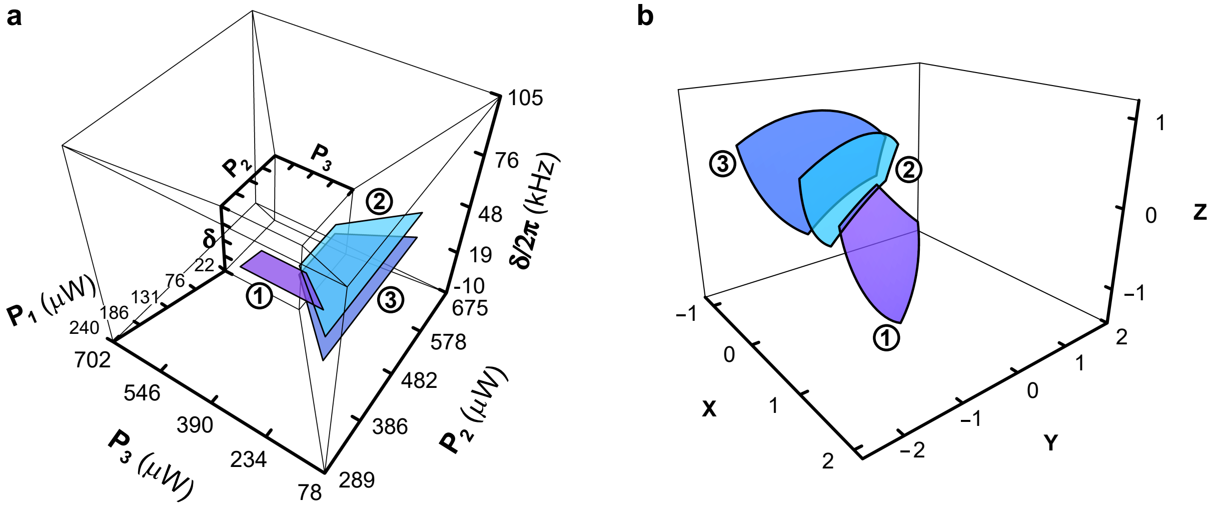

Supplementary Fig. 1. The sheets used to form the 2D control spaces shown in Figs. 3a, 4a, 5a. Sheets ② and ③ are joined to make $\mathcal{B}^{(1)}$ in Fig. 3a. Sheets ①, ② and ③ are joined to make $\mathcal{B}^{(2)}$ in Fig. 4a. Sheets ① and ② are joined to make $\mathcal{B}^{(3)}$ in Fig. 5a. **a** The three sheets, shown in a rectilinear stereographic projection of the hypersurface $\mathcal{S}$. **b** The same sheets, shown in the stereographic projection used in Figs. 3(b,c), 4(b,c), 5(b,c).
